# Supplementary material for: Mature interneuron subtypes arise from distinct spatial and temporal subdomains within the caudal ganglionic eminence
Source: bioRxiv. 2025 Jul 29:2025.07.28.667082. Preprint. [Version 1] doi: 10.1101/2025.07.28.667082 (PMC12443043; doi:10.1101/2025.07.28.667082)
Supplement: 1 [file NIHPP2025.07.28.667082V1-supplement-1.pdf]

bioRxiv preprint doi: https://doi.org/10.1101/2025.07.28.667082; this version posted July 29, 2025. The copyright holder for this preprint (which was not certified by peer review) is the author/funder. This article is a US Government work. It is not subject to copyright under 17 USC 105 and is also made available for use under a CC0 license.

494

Figure S1

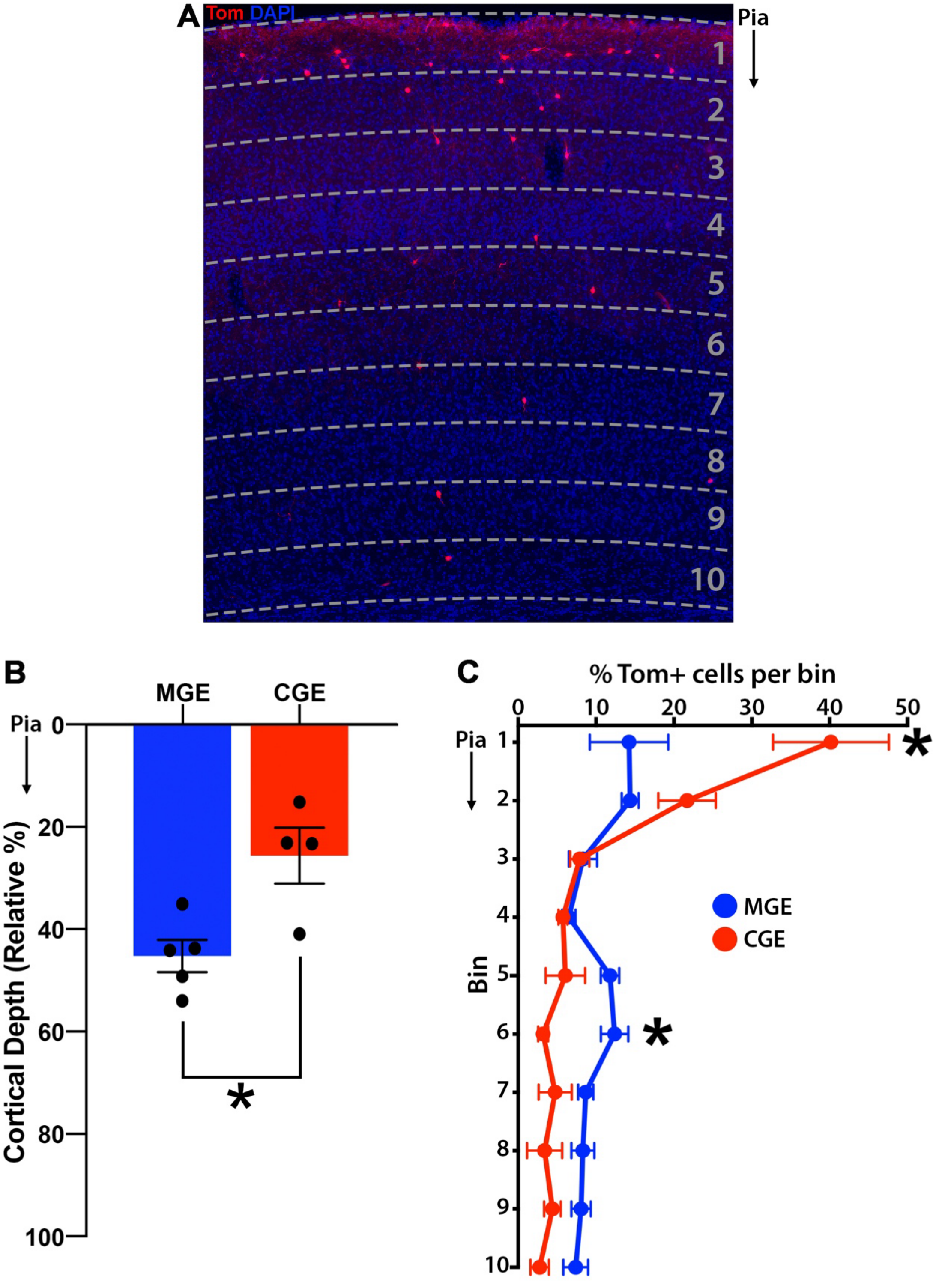

495

bioRxiv preprint doi: https://doi.org/10.1101/2025.07.28.667082; this version posted July 29, 2025. The copyright holder for this preprint (which was not certified by peer review) is the author/funder. This article is a US Government work. It is not subject to copyright under 17 USC 105 and is also made available for use under a CC0 license.

Figure S1. Transplanted CGE-derived cells are biased towards superficial layers. **A.** Example of grafted Tom+ cells with equidistant bins spanning from the pial surface to the layer VI boundary. **B.** Bar graph depicting average depth of grafted Tom+ MGE and CGE cells, ranging from 0 at the pia surface to 100 at the layer VI-subplate boundary. * $p < 0.05$. **C.** Line graph depicting the percent of grafted Tom+ MGE and CGE cells in 10 equivalent size bins spanning from the pia (Bin 1) to the white matter boundary below layer VI (Bin 10).

bioRxiv preprint doi: https://doi.org/10.1101/2025.07.28.667082; this version posted July 29, 2025. The copyright holder for this preprint (which was not certified by peer review) is the author/funder. This article is a US Government work. It is not subject to copyright under 17 USC 105 and is also made available for use under a CC0 license.

Figure S2

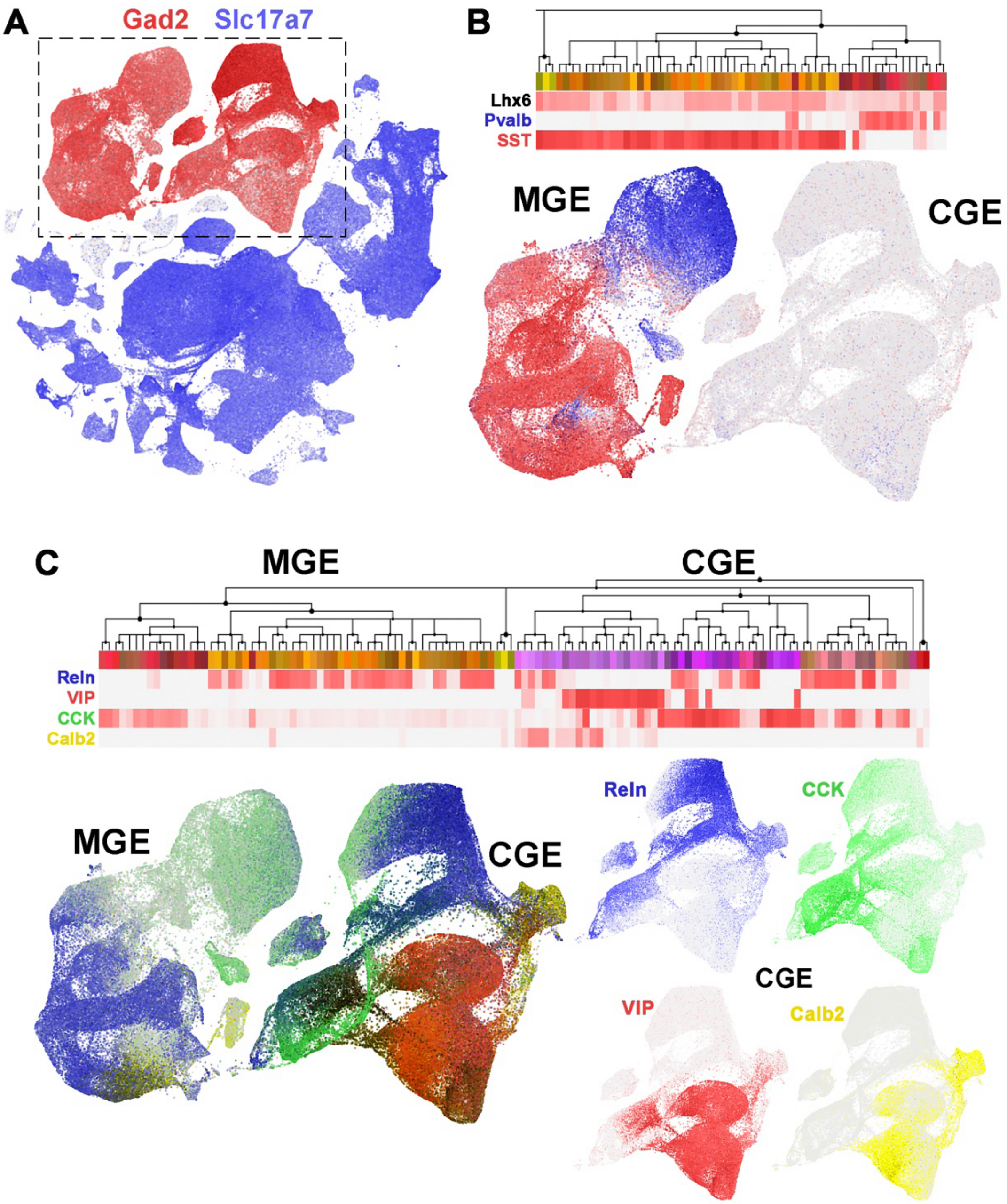

bioRxiv preprint doi: https://doi.org/10.1101/2025.07.28.667082; this version posted July 29, 2025. The copyright holder for this preprint (which was not certified by peer review) is the author/funder. This article is a US Government work. It is not subject to copyright under 17 USC 105 and is also made available for use under a CC0 license.

Figure S2. Markers of mature CGE-derived interneuron subtypes. All heatmaps and scatterplots are taken and adapted from the Allen Brain Map Cell Types Database: RNA-Seq Data (portal.brain-map.org/atlases-and-data/rnaseq), specifically the mouse whole cortex & hippocampus –10x Genomics with 10x smart-sequencing taxonomy (47). **A.** Scatterplot of all cells in the dataset; inhibitory and excitatory neurons can be cleanly segregated by expression of Gad2 and Slc17a7, respectively. **B.** Heatmap (top) and scatterplot (bottom) demonstrating MGE-derived interneurons can be cleanly segregated into 2 cardinal subclasses expressing either Pvalb (PV) or SST. **C.** Heatmap (top) and scatterplot (bottom) showing expression of 4 markers used in this study to define CGE-derived interneurons, highlighting the complimentary expression patterns of VIP (red) and Reln (blue), and CCK (green) and CR (yellow).

bioRxiv preprint doi: https://doi.org/10.1101/2025.07.28.667082; this version posted July 29, 2025. The copyright holder for this preprint (which was not certified by peer review) is the author/funder. This article is a US Government work. It is not subject to copyright under 17 USC 105 and is also made available for use under a CC0 license.

Figure S3

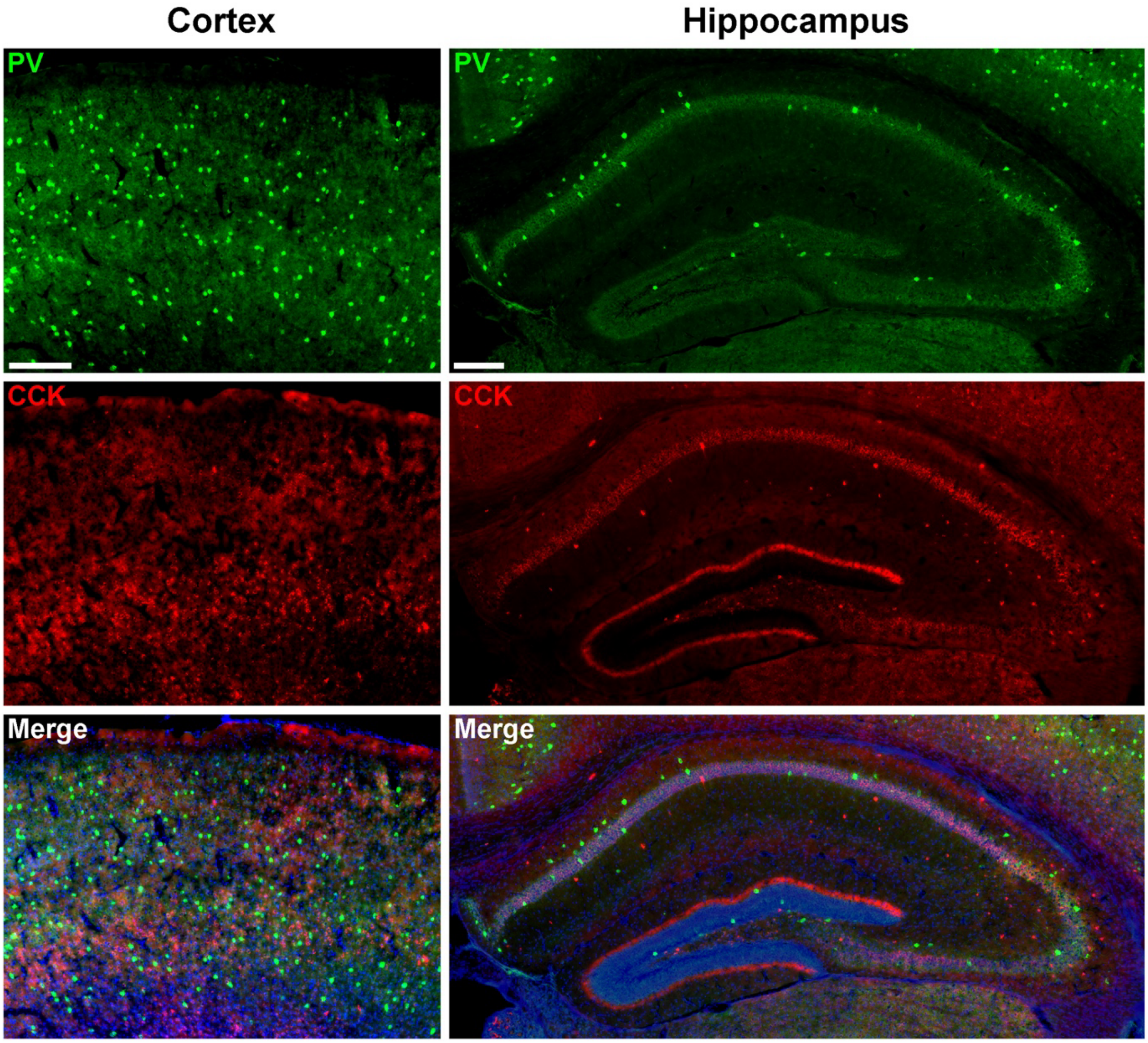

Figure S3. MGE-derived PV+ interneurons do not express CCK protein. Representative images through the cortex and hippocampus showing lack of colocalization between PV and CCK proteins. Scale bars = 100 μm.

bioRxiv preprint doi: https://doi.org/10.1101/2025.07.28.667082; this version posted July 29, 2025. The copyright holder for this preprint (which was not certified by peer review) is the author/funder. This article is a US Government work. It is not subject to copyright under 17 USC 105 and is also made available for use under a CC0 license.

Figure S4

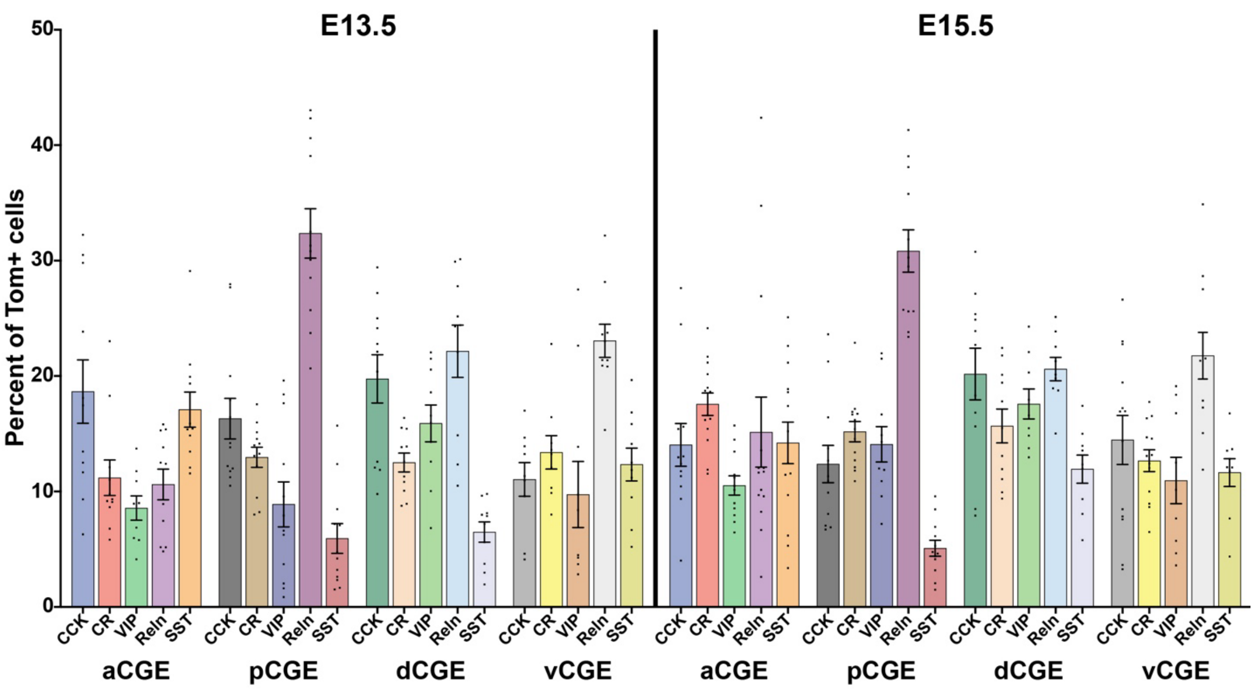

Figure S4. All datapoints used for analysis. Graph depicting all datapoints for each immunostained cell maker CCK, CR, VIP, Reln (Reln+/SST-) and SST (Reln+/SST+) for all 8 experimental conditions; 4 CGE subdomains at E13.5 (left) and E15.5 (right).

bioRxiv preprint doi: https://doi.org/10.1101/2025.07.28.667082; this version posted July 29, 2025. The copyright holder for this preprint (which was not certified by peer review) is the author/funder. This article is a US Government work. It is not subject to copyright under 17 USC 105 and is also made available for use under a CC0 license.

SUPPLEMENTARY TABLE LEGENDS

Supplementary Table 1. Statistics for E13.5 vs. E15.5 timepoints. Unpaired t-tests were used to compare E13.5 vs. E15.5 for all CGE regions for all cell types, and for all regions combined for each cell type (last column). P-values presented for each t-test. Significant results are bold, results with a trend that did not reach significance ($0.10 > p > 0.05$) are highlighted in red. ** $p < 0.01$.

Supplementary Table 2. Statistics for all E13.5 transplants. One-way ANOVA was used to compare all 4 brain regions, followed by Tukey's multiple comparison t-test between 2 regions. F and P values given for each ANOVA (right column), and adjusted P-value presented for each t-test. Significant results are bold, results with a trend that did not reach significance ($0.10 > p > 0.05$) are highlighted in red. * $p < 0.05$, ** $p < 0.01$, *** $p < 0.0005$, **** $p < 0.0001$.

Supplementary Table 3. Statistics for all E15.5 transplants. One-way ANOVA was used to compare all 4 brain regions, followed by Tukey's multiple comparison t-test between 2 regions. F and P values given for each ANOVA (right column), and adjusted P-value presented for each t-test. Significant results are bold, results with a trend that did not reach significance ($0.10 > p > 0.05$) are highlighted in red. * $p < 0.05$, ** $p < 0.01$, *** $p < 0.0005$, **** $p < 0.0001$.

Supplementary Table 1 – Statistics for E13.5 vs E15.5 comparisons

**E13.5 vs. E15.5**	**aCGE**	**pCGE**	**dCGE**	**vCGE**	**All regions combined**
**CCK**	NS<br>0.172	NS<br>0.113	NS<br>0.892	NS<br>0.243	NS<br>0.317
**CR**	******<br>**0.001**	NS<br>0.087	NS<br>0.073	NS<br>0.666	******<br>**0.001**
**VIP**	NS<br>0.156	NS<br>0.055	NS<br>0.430	NS<br>0.739	NS<br>0.123
**Reln+/SST-**	NS<br>0.224	NS<br>0.593	NS<br>0.556	NS<br>0.650	NS<br>0.866
**Reln+/SST+**	NS<br>0.249	NS<br>0.570	******<br>**0.002**	NS<br>0.716	NS<br>0.801

Supplementary Table 2 – Statistics for spatial E13.5 comparisons

E13.5	aCGE vs. pCGE	aCGE vs. dCGE	aCGE vs. vCGE	pCGE vs. dCGE	pCGE vs. vCGE	dCGE vs. vCGE	ANOVA comparing all 4 regions
**CCK**	NS<br>0.850	NS<br>0.982	NS<br>0.086	NS<br>0.636	NS<br>0.327	*****<br>**0.039**	*****<br>**P value = 0.042**<br>F = 3.008
**CR**	NS<br>0.691	NS<br>0.855	NS<br>0.590	NS<br>0.992	NS<br>0.995	NS<br>0.957	NS<br>P value = 0.600<br>F = 0.637
**VIP**	NS<br>0.999	NS<br>0.082	NS<br>0.979	NS<br>0.071	NS<br>0.990	NS<br>0.162	*****<br>**P value = 0.049**<br>F = 2.874
**Reln+/SST-**	********<br>**<0.0001**	*******<br>**0.0005**	*******<br>**0.0002**	******<br>**0.0019**	******<br>**0.0051**	NS<br>0.987	********<br>**P value < 0.0001**<br>F = 24.52
**Reln+/SST+**	********<br>**<0.0001**	********<br>**<0.0001**	NS<br>0.072	NS<br>0.990	******<br>**0.007**	*****<br>**0.021**	********<br>**P value < 0.0001**<br>F = 16.57

Supplementary Table 3 – Statistics for spatial E15.5 comparisons

**E15.5**	**aCGE vs. pCGE**	**aCGE vs. dCGE**	**aCGE vs. vCGE**	**pCGE vs. dCGE**	**pCGE vs. vCGE**	**dCGE vs. vCGE**	**ANOVA comparing all 4 regions**
**CCK**	NS<br>0.933	NS<br>0.152	NS<br>0.999	*<br>**0.043**	NS<br>0.870	NS<br>0.189	NS<br>P value = 0.051<br>F = 2.797
**CR**	NS<br>0.367	NS<br>0.604	**<br>**0.008**	NS<br>0.989	NS<br>0.333	NS<br>0.221	*<br>**P value = 0.015**<br>F = 3.842
**VIP**	NS<br>0.260	**<br>**0.005**	NS<br>0.996	NS<br>0.336	NS<br>0.431	*<br>**0.016**	**<br>**P value = 0.004**<br>F = 5.226
**Reln+/SST-**	****<br>**<0.0001**	NS<br>0.401	NS<br>0.237	*<br>**0.032**	NS<br>0.069	NS<br>0.990	***<br>**P value = 0.0002**<br>F = 8.203
**Reln+/SST+**	****<br>**<0.0001**	NS<br>0.663	NS<br>0.570	**<br>**0.009**	*<br>**0.014**	NS<br>0.999	***<br>**P value = 0.0001**<br>F = 8.852
